# 254. Genomic diversity of Gram-negative Bacilli from Bloodstream Infections in Hospitalized Children

**DOI:** 10.1093/ofid/ofad500.326

**Published:** 2023-11-27

**Authors:** Jennifer Blumenthal, Arolyn Conwill, Patrick McGann, Thomas J Sandora, Tami Lieberman, Gregory P Priebe

**Affiliations:** Boston Children's Hospital, Boston, Massachusetts; Massachusetts Institute of Technology, Boston, Massachusetts; Walter Reed Army Institute, Silver Spring, Maryland; Boston Children's Hospital, Boston, Massachusetts; Massachusetts Institute of Technology, Boston, Massachusetts; Boston Children's Hospital, Boston, Massachusetts

## Abstract

**Background:**

Gram-negative bloodstream infection (BSI) in hospitalized children is associated with significant morbidity and mortality. It is unclear whether bacterial clonality of isolates during BSI in a unique patient is associated with clinical characteristics such as immunocompromise. We hypothesized that genomic diversity of BSI isolates would be higher in immunocompromised patients due to mucosal barrier injury and therefore larger translocation bottleneck.

**Methods:**

We prospectively enrolled patients with a central venous catheter who were hospitalized at Boston Children’s Hospital and had a positive blood culture that grew *E. coli*, *Klebsiella spp*., or *Pseudomonas aeruginosa*. An aliquot from the blood culture bottle was plated on agar, and at least 24 colonies were picked randomly and subjected to whole-genome sequencing (WGS). Patient-specific reference genomes were constructed using long-read sequencing of at least 1 isolate on the PacBio platform. All isolates were sequenced using Illumina (MiSeq or NextSeq). Single nucleotide variant (SNV) calls were made using the WideVariant pipeline (github). Clinical characteristics were obtained retrospectively from the electronic medical record. We defined immunocompromise as neutropenia (ANC< 500) on the day of BSI or prior solid organ or stem cell transplant. The proportion of BSI episodes with clonal blood isolates (defined as 0 SNV) was compared between immunocompromised and non-immunocompromised patients using Fisher’s exact test.

**Results:**

We enrolled 34 patients (median age 2.33 years, IQR 0.8-3.8 years), 25 having BSI from Enterobacterales (11 *E. coli*, 14 *Klebsiella spp*.) and 9 from *Pseudomonas aeruginosa*. Immunocompromised patients had a significantly higher proportion of Enterobacterales BSI episodes with clonal isolates compared with non-immunocompromised patients [13/18 (72%) vs. 1/7 (14%) respectively, *P*=0.021). This was not seen with Pseudomonas BSI, which had clonal isolates in 3/5 (60%) vs. 2/4 (50%) respectively (*P*=1).

**Conclusion:**

Contrary to our hypothesis, immunocompromised patients were more likely to have clonal Enterobacterales BSI isolates compared with non-immunocompromised patients, perhaps due to lower host pressures for bacterial population diversification in immunocompromised patients.

**Disclosures:**

**Arolyn Conwill, PhD**, Day Zero Diagnostics: current employee|Day Zero Diagnostics: Stocks/Bonds

